# Effects of oral health-related quality of life on total mortality: a prospective cohort study

**DOI:** 10.1186/s12903-023-03451-8

**Published:** 2023-10-03

**Authors:** Nishiki Arimoto, Rumi Nishimura, Teruo Kobayashi, Mayuka Asaeda, Toru Naito, Masaaki Kojima, Osami Umemura, Makoto Yokota, Nobuhiro Hanada, Takashi Kawamura, Kenji Wakai, Mariko Naito

**Affiliations:** 1 Department of Oral Epidemiology, Hiroshima University Graduate School of Biomedical and Health Sciences, Hiroshima, Japan; 2 Department of Dentistry and Oral Surgery, Asahi General Hospital, Asahi, Chiba Japan; 3 Department of Oral Health, Faculty of Health Sciences, Kobe Tokiwa University, Kobe, Hyogo Japan; 4 Section of Geriatric Dentistry, Department of General Dentistry, Fukuoka Dental College, Fukuoka, Japan; 5 Aichi Dental Association, Nagoya, Aichi Japan; 6 Yokota Makoto Dental Clinic, Fukuoka, Japan; 7 Photocatalysis International Research Center, University of Shanghai for Science and Technology, Shanghai, P.R. China; 8 Department of Preventive Services, Kyoto University School of Public Health, Kyoto, Japan; 9 Department of Preventive Medicine, Nagoya University Graduate School of Medicine, Nagoya, Aichi Japan

**Keywords:** Oral health-related quality of life, OHQOL, Quality of life, QOL, Total mortality, Oral health

## Abstract

**Background:**

The effects of oral health on mortality have been reported; however, the association between mortality and Oral Health-Related Quality of Life (OHQOL) is unknown. We investigated the effect of OHQOL on total mortality in a cohort consisting of dentists.

**Methods:**

In this cohort study, we analyzed data from the Longitudinal Evaluation of Multi-phasic, Odonatological and Nutritional Associations in Dentists study. We conducted a baseline survey of general and oral health factors. We called for 31,178 participants and collected responses from 10,256 participants. We followed up with 10,114 participants (mean age ± standard deviation, 52.4 ± 12.1 years; females, 8.9%) for 7.7 years, until March 2014, to determine the average total mortality. OHQOL was assessed using the General Oral Health Assessment Index (GOHAI). The total score was divided into quartiles (Q1 ≤ 51.6, Q2 = 51.7–56.7, Q3 = 56.8–59.9, and Q4 = 60.0), with higher GOHAI scores indicating better OHQOL (score range, 12–60). The association between OHQOL and total mortality was analyzed using the Cox proportional hazards model.

**Results:**

We documented 460 deaths. Males with low GOHAI scores possessed a remarkably high risk of total mortality. The multivariate adjusted-hazard ratios (aHRs), were 1.93 (95% confidence interval [CI], 1.07 − 3.48) for Q1, 1.69 (95% CI, 0.90 − 3.17) for Q2, and 0.65 (95% CI, 0.29 − 1.46) for Q3, relative to Q4 (trend *p* = 0.001). The aHRs in the multivariate model with all background variables were 1.69 (95% CI, 1.15–2.46) for Q1, 1.53 (95% CI, 1.04–2.27) for Q2, and 1.09 (95% CI, 0.71–1.70) for Q3, relative to Q4 (trend* p* = 0.001). In females, there was no significant association between the quartiles, in both the multivariate-adjusted model (trend* p* = 0.52) and multivariate-adjusted model with all background variables (trend *p* = 0.79).

**Conclusions:**

A lower OHQOL indicated an increased risk of total mortality in dentists. OHQOL may be used as an indicator for selecting treatment plans and personalized care interventions, thus contributing to increased healthy life expectancy.

**Trial registration:**

Aichi Cancer Center, Nagoya University Graduate School of Medicine, and Hiroshima University (Approval numbers: 33, 632–3, 8–21, and E2019-1603).

**Supplementary Information:**

The online version contains supplementary material available at 10.1186/s12903-023-03451-8.

## Background

The average life expectancy is increasing in many developed countries. In 2018, the average life expectancy of people in Japan was the highest worldwide at 87.57 years in females and 81.47 years in males [1]. Demographic factors [2], oral health status [3, 4], nutritional status [5], life, and environment [6] factors have been shown to affect mortality. Health-related quality of life (HQOL) has also been identified among these factors [7].

HQOL is an assessment tool that provides an accurate indication of a patient’s health status and outcomes [8]. It has been associated with an improvement in the disease-specific prognosis and the prediction of patient outcomes [8–10]. Additionally, mortality rates in patients with chronic kidney disease have been associated with low HQOL [11]. Further, quality of life (QOL) is used to assess the effects of clinical treatment [12]. Therefore, QOL assessments are useful for examining the study endpoints, including death [13]. Regarding QOL, systemic HQOL can be assessed in general clinical practice, and the assessment of objective indices, such as the body mass index and blood pressure, can identify individuals who are at high risk of adverse health outcomes or mortality [12].

Oral health has been associated with mortality [14–18]. For example, the loss of multiple teeth results in occlusal collapse and undernutrition, as the masticatory ability declines [16]. The relationship between the number of missing teeth and the risk of death from pneumonia has been reported [15]. Moreover, a lower number of remaining teeth has been associated with an increased risk of all-cause mortality and lung cancer [17]. Poor oral health on admission is a predictor of mortality among geriatric patients in acute care hospitals [18].

Oral HQOL (OHQOL) is a subjective indicator that is used to estimate the extent to which oral health disorders affect the physical functions and psychosocial health of a patient [19]. While many studies have reported the effects of oral health on OHQOL [20–22], few studies have investigated the association between total mortality and OHQOL. Therefore, this prospective cohort study including Japanese dentists investigated the association between OHQOL and total mortality.

## Methods

### Data set

This study was conducted using data from the Longitudinal Evaluation of Multi-phasic, Odontological, and Nutritional Associations in Dentists (LEMONADE) cohort study [15, 23, 24], which included dentists who are members of the Japan Dental Association (JDA).

From 2001 to 2006, a baseline survey was conducted on the study participants’ lifestyle, medical history, general health and oral health factors, including the number of missing teeth, and current drug use status. Further, depressive tendencies in participants were assessed using the 12-item General Health Questionnaire (GHQ-12). Of the 58,792 participants who received questionnaires from 46 prefectures, 21,272 participants responded (response rate: 36.2%). The OHQOL assessment using GOHAI was conducted with 31,178 participants from some prefectures (20/46 prefectures) and 10,256 responded (response rate: 32.9%). The statistical analysis of this study included 10,114 participants. The ages of the survey respondents ranged from 26 to 98 years or more, with the highest percentage of both male and female respondents aged 40–49 years.

### OHQOL assessment

The OHQOL of the study participants was assessed using the GOHAI, which was originally developed for oral health assessments of the older population. However, its effectiveness has also been reported in younger individuals [19]. It was translated into Japanese by Naito et al. [25], and the national standard value (total sample: average 53.1 ± 7.0, range 25–60) was released to the public in 2006 (https://oral-epi.jpn.org).

GOHAI consists of 12 questions. The participants responded to the questions in the form of scores on a Likert scale (1 to 5 points). The response options were as follows: all the time (1 point), most of the time (2 points), some of the time (3 points), a little of the time (4 points), and never (5 points). The total scores ranged from 12 to 60 points, and a higher score indicated a better OHQOL.

### Follow-up and outcome

The participants were followed up until March 2014. After obtaining informed consent from all the participants, the mortality and morbidity data were collected via the fraternal insurance program of the JDA. The outcome was total mortality. However, there were certain exceptions because the completion dates were different (December 2010, March 2012, and March 2015 in some late-starting prefectures). The fraternal insurance program provides insurance benefits after completing the prescribed procedures for the mortality or morbidity of a JDA member.

The cause of death was determined from the information on the certificate. Since this fraternal insurance was independent of health insurance, the mortality data of the JDA members could be determined from this program, even if they had changed their health insurance. Participants leaving the JDA or refusing further follow-up were excluded from the study. This study has been reported as per the Strengthening the Reporting of Observational Studies in Epidemiology (STROBE) guidelines.

### Statistical analysis

The quartiles of the total scores for the 12 items of the GOHAI were calculated. A Q4 quartile was regarded as high OHQOL, and Q1 as low OHQOL (Q1 ≤ 51.6, Q2 = 51.7 − 56.7, Q3 = 56.8–59.9, and Q4 = 60.0).

Data pertaining to the demographic characteristics of the participants are presented as means, standard deviations, and percentages, using descriptive statistics. To assess the history of systemic disease among the participants, strokes, myocardial infarction, and cancer were grouped to create a category for any prevalence (with or without history). The participants were categorized according to their smoking status as never, former, or current smokers. Similarly, they were categorized according to their alcohol consumption status as never, former, and current drinkers.

The number of missing teeth and sleeping time were considered continuous variables. For vigorous physical activity, we enquired about the participants’ exercise duration within the past week, with jogging, mountain biking, tennis, squash, swimming, rap dance, and aerobics as examples. The participants were categorized based on the presence or absence of vigorous physical activity. According to the drug use status, they were classified into the following groups: with or without use. In the case of drug use, the drugs used were classified as for medication (hypertension, hypercholesterolemia, or gout or hyperuricemia), diabetes (oral medication or insulin self-injection) [26], and sleeping (at least once a week) [27–29]. Diabetes was associated with the risk of all-cause death [30]. The use of sleeping pills in depressed patients is well known, and sleep disturbances have been reported to affect mortality risk [31]. Therefore, in this study, medication status was categorized and included in the analysis. The GHQ-12 was set at a cutoff value of ≥ 4 points.

Sex-specific and age-specific characteristics, as well as associations between GOHAI score quartiles and variables that affected oral health, were evaluated using one-way analysis of variance. The adjusted hazard ratios (aHRs) and 95% confidence interval (95% CI) for OHQOL and total mortality were calculated using a Cox proportional hazards model. First, after analyzing the age-sex adjustment model, the background variables were analyzed using a multivariate model for continuous (age, sex, number of missing teeth, and sleeping time) and categorical variables (medication use status [with use], diabetes drug use [with use], sleeping pills use [with use], history of systemic disease [with history], vigorous physical activity [yes], smoking status [never, former, or current], alcohol consumption status [never, former, or current], and GHQ score [≥ 4 points]). Additionally, multivariate models were created both without (multivariate-adjusted model 1) and with adjustment for the number of missing teeth (multivariate-adjusted model 2). Linear trends were estimated by adding variables to the analytical model that provided new scores of 0–3 to the GOHAI scores quartiles and calculating the *p*-value for trends in males and females to examine the dose–response relationship between OHQOL and the risk of total mortality. The proportional hazard assumption was graphically assessed via a log–log plot, and we confirmed that the proportional hazards assumption was not violated.

Furthermore, our analysis was limited to those with data related to the General Health (GH) section of the SF-36 (*n* = 8,562). Similar to our main analysis, the examination was performed via the Cox proportional hazards model, which was adjusted for different background variables. The GOHAI scores quartiles were created using the GOHAI scores of the participants who had also provided the GH data of the SF-36. Additionally, we conducted a multivariate analysis incorporating a GH score with continuous variables. The GH section comprised five items, all of which were answered using a Likert scale (1–5 points). Subscale scores (range: 0–100) were calculated from the response data according to the SF-36 manual and subsequently used in the analysis.

The statistical significance level was set at *p* < 0.05. All statistical analyses were performed using IBM SPSS Statistics for Windows version 25 (IBM Corp., SPSS, Japan Inc., Tokyo, Japan).

## Results

The mean age ± standard deviation of the participants was 52.4 ± 12.1 (range, 26–98) years, with females accounting for 8.0% (*n* = 903) of the total study population. During an average follow-up period of 7.7 years, we documented 460 deaths that were indicative of total mortality, with a person-year of 45.481/1,000. The mean GOHAI score of the participants was 54.6 ± 6.2. According to the age groups, participants aged 26–29 years had the highest GOHAI scores (20 s: 57.3 ± 3.9, 30 s: 56.0 ± 5.2, 40 s: 55.5 ± 5.4, 50 s: 54.5 ± 6.1, 60 s: 53.4 ± 6.8, 70 s: 51.5 ± 7.7, 80 s: 50.8 ± 7.9, and 90 s: 52.4 ± 6.3).

Table 1 shows the baseline characteristics of the participants according to the GOHAI score quartiles. Q4, which indicated a better OHQOL, was a characteristic of young participants who had no history of systemic diseases, were not current smokers, had a smaller number of missing teeth, had a lower GHQ score of ≥ 4 points, performed vigorous physical activity, and did not use drugs (*p* < 0.001). The male participants generally had a smaller number of missing teeth, were current drinkers and current smokers, performed vigorous physical activity, and used drugs for medication and diabetes more frequently than female participants (*p* < 0.001, < 0.05, respectively). The characteristics of OHQOL based on the sex of the participants are contained in Additional files 1 and 2 (Baseline characteristics of male and female participants by GOHAI score quartile.

**Table 1:** Baseline characteristics of the participants by GOHAI score quartile

Baseline characteristics	GOHAI score quartile				
	Q1 (≤ 51.6)	Q2 (51.7–56.7)	Q3 (56.8–59.9)	Q4 (60.0)	*p*
Number of participants^a^	2491	2516	2534	2573	
Age (years, mean ± SD)	56.6 ± 13.1	52.3 ± 11.8	50.9 ± 11.3	49.9 ± 11.1	< 0.001**
Female sex (%)	7.8	9.8	9.4	8.8	0.08
History of systemic diseases, %^b^	11.8	6.8	5.8	6.5	< 0.001**
Current smoker (%)	31.4	27.7	27.8	25	< 0.001**
Current drinker (%)	69.1	71.6	74	73.8	< 0.001**
Number of missing teeth (mean ± SD)	6.2 ± 7.9	3.1 ± 5.8	2.2 ± 4.5	1.7 ± 4.1	< 0.001**
Sleeping time (mean, hour)	6.9	6.8	6.9	6.9	0.38
Vigorous physical activity (%)^c^	19.9	24	25.3	27.4	< 0.001**
Medication use (with use, %)^d^	27.7	22.4	20.2	18.3	< 0.001**
Sleeping pills use (with use, %)^e^	6.3	4	2.3	2.6	< 0.001**
Diabetes drug use (with use, %)^f^	6.4	4.5	3.4	3.3	< 0.001**
GHQ-12 score (≥ 4 points, %)^g^	35.7	27.6	22.8	18.1	< 0.001**

*GOHAI* General Oral Health Assessment Index, *SD* standard deviation

^a^Surveyed in 20 of the 46 prefectures of the Japanese Dental Association

^b^History of systemic diseases: cancer, myocardial infarction, and stroke

^c^Vigorous physical activity (%): Jogging, mountain biking, tennis, squash, swimming, rap dance, and aerobics as examples

^d^Drugs for hypertension, hypercholesterolemia, or gout or hyperuricemia

^e^Drugs for sleeping (at least once a week)

^f^Drugs for diabetes (oral medication or insulin self-injection)

^g^GHQ score: The General Health Questionnaire-12 (GHQ-12)

^*^p < 0.05; **p < 0.001

Male participants who had low GOHAI scores at baseline were at a significantly higher risk of total mortality (Table 2). Further, in the multivariate-adjusted model 1, aHRs derived from Cox regression analysis were 1.93 (95% CI, 1.07 − 3.48) for Q1, 1.69 (95% CI, 0.90 − 3.17) for Q2, and 0.65 (95% CI, 0.29 − 1.46) for Q3, relative to Q4 (trend *p* = 0.001). In the multivariate-adjusted model 2, aHRs were 1.69 (95% CI, 1.15–2.46) for Q1, 1.53 (95% CI, 1.04–2.27) for Q2, and 1.09 (95% CI, 0.71–1.70) for Q3, relative to Q4 (trend *p* = 0.001). In females, there were no differences between the quartiles in both bivariate and multivariate model analyses (Table 3). Furthermore, the *p*-value for trend was not significant in either the multivariate-adjusted model 1 (trend *p* = 0.52) or in the multivariate-adjusted model 2 (trend *p* = 0.79).

**Table 2:** aHR for total mortality based on male sex according to the GOHAI score quartile

			Age-and sex-adjusted		Multivariate-adjusted model 1		Multivariate-adjusted model 2	
GOHAI score quartile by sex	n (%)	Death (%)	aHR^a^	95% CI	aHR^b^	95% CI	aHR^c^	95% CI
Male sex								
Q4	2347 (25.5)	56 (13.1)	1		1		1	
Q3	2297 (24.9)	60 (14.1)	0.97	0.67—1.40	0.65	0.29—1.46	1.09	0.71—1.70
Q2	2270 (24.6)	108 (25.3)	1.51	1.09—2.09	1.69	0.90—3.17	1.53	1.04—2.27
Q1	2297 (24.9)	203 (47.5)	1.76	1.31—2.37	1.93	1.07—3.48	1.69	1.15—2.46
Total	9211	427	*p* for trend = < 0.001**		*p* for trend = 0.001**		*p* for trend = 0.001**	

*aHR* adjusted hazard ratio, *GOHAI* General Oral Health Assessment Index, *CI* confidence interval

^a^Adjusted for age and sex

^b^Adjusted for continuous (age, sex, and sleeping time) and categorical variables (medication use status [with use], sleeping pills use [with use], diabetes drug use [with use], GHQ score [≥ 4 points], history of systemic disease [with history], vigorous physical activity [yes], smoking status [never, former, or current], and drinking status [never, former, or current])

^c^Adjusted for continuous (age, sex, number of missing teeth, and sleeping time) and categorical variables (medication use status [with use], sleeping pills use [with use], diabetes drug use [with use], GHQ score [≥ 4 points], history of systemic disease [with history], vigorous physical activity [yes], smoking status [never, former, or current], and drinking status [never, former, or current])

^*^p < 0.05; **p < 0.001

**Table 3:** aHR for total mortality based on female sex according to the GOHAI score quartile

			Age-and sex-adjusted		Multivariate-adjusted model 1		Multivariate-adjusted model 2	
GOHAI score quartile by sex	n (%)	Death (%)	aHR^a^	95% CI	aHR^b^	95% CI	aHR^c^	95% CI
Female sex								
Q4	226 (25.0)	7 (21.2)	1		1		1	
Q3	237 (26.2)	6 (18.2)	0.68	0.23—2.01	2.16	0.21—21.81	0.91	0.22—3.82
Q2	246 (27.2)	6 (18.2)	0.58	0.20—1.74	2.02	0.20—20.07	0.74	0.16—3.44
Q1	194 (21.5)	14 (42.4)	0.85	0.34—2.15	2.11	0.29—15.28	1.17	0.33—4.17
Total	903	33	*p* for trend = 0.85		*p* for trend = 0.52		*p* for trend = 0.79	

*aHR* adjusted hazard ratio, *GOHAI* General Oral Health Assessment Index, *CI* confidence interval

^a^Adjusted for age and sex

^b^Adjusted for continuous (age, sex, and sleeping time) and categorical variables (medication use status [with use], sleeping pills use [with use], diabetes drug use [with use], GHQ score [≥ 4 points], history of systemic disease [with history], vigorous physical activity [yes], smoking status [never, former, or current], and drinking status [never, former, or current])

^c^Adjusted for continuous (age, sex, number of missing teeth, and sleeping time) and categorical variables (medication use status [with use], sleeping pills use [with use], diabetes drug use [with use], GHQ score [≥ 4 points], history of systemic disease [with history], vigorous physical activity [yes], smoking status [never, former, or current], and drinking status [never, former, or current])

^*^p < 0.05; **p < 0.001

We performed an analysis regarding the GH of the HQOL. The results of this can be found in Additional files 3 and 4 (total aHRs examined based on the male and female sex categories according to the GOHAI score quartiles, while considering the presence of General Health data from the HQOL dataset). In the analyses where the GH score was added as an adjustment variable, the association between OHQOL and the mortality risk among males was attenuated but continued to demonstrate considerable values across both models. Specifically for males, the aHRs in the multivariate-adjusted model 1 were 1.54 (95% CI, 1.03 − 2.31) for Q1, 1.27 (95% CI, 0.82 − 1.97) for Q2, and 0.93 (95% CI, 0.56 − 1.57) for Q3, relative to Q4 (trend *p* = 0.01). Furthermore, the aHRs in the multivariate-adjusted model 2 were 1.48 (95% CI, 0.98–2.24) for Q1, 1.26 (95% CI, 0.81–1.95) for Q2, and 0.93 (95% CI, 0.56–1.57) for Q3, relative to Q4 (trend *p* = 0.02). On the contrary, among females, there was no substantial association between OHQOL and risk of mortality.

## Discussion

In this study, OHQOL was positively associated with the risk of total mortality in the Japanese dentist population. The results of the stratified analysis demonstrated that a lower OHQOL significantly increased the risk of total mortality in males. This association was maintained after adjusting for the potential confounders that affected oral health.

Similar to the objective indicators of oral health, OHQOL can predict the risk of total mortality. Regarding the relationship between oral health and mortality, edentulous patients who did not use dentures at baseline had approximately twice the mortality rate after the follow-up than those who had 20 or more teeth [32]. In addition, dentition status, age, and sex are known risk factors for high mortality [32]. Tooth loss is known to significantly increase the risk of all-cause or cause-specific mortality [3].

OHQOL can identify the impact of oral health on aspects of daily living [33]. In a study investigating the relationship between HQOL and OHQOL in patients after heart transplantation and in those with heart failure, OHQOL was reported to be associated with the Physical Component Summary and Mental Component Summary in HQOL [34]. To examine the impact of the oral-related factors of HQOL on the mortality risk, multivariate model analyses were conducted for participants who provided data on the GH of the HQOL. Although the adjustment for GH did attenuate the association, the considerable link between OHQOL and the risk of mortality persisted for males. This suggests that the OHQOL factors could potentially hold predictive value for mortality outcomes while controlling for HQOL. OHQOL is a component of HQOL, and a bidirectional relationship is present between the two indices [35]. The results obtained from the QOL index can help in the selection of interventions and care plans that are specific to the identified risks, thus increasing their effectiveness [36]. Therefore, they can be expected to improve the health of individuals and decrease mortality.

A comparison was made between the characteristics of the Japanese dental population and those of the participants in this study. Data concerning the Japanese dental population was obtained from the 2004 Ministry of Health, Labor, and Welfare's Summary Survey of Doctors, Dentists, and Pharmacists. When comparing this study’s participant population with the Japanese dental population (2004), we identified that the participant population had a higher proportion of males and a smaller proportion of females (Japanese dental population: males 81%, females 19%; participant population: males 91%, females 9%). In terms of age distribution by sex, differences were observed between the two populations, particularly among females. Within the female subgroup, most individuals were in the younger age ranges (20 s–40 s) in the Japanese dental population. Conversely, the participant population exhibited a more substantial representation in the 40 s–50 s age range, indicating a distribution difference. For males, the overall distribution of the two groups was similar. However, there was a difference in terms of the proportion within the 20 s age group, which was higher in the Japanese dental population than in the participant population [37].

Our study demonstrated that OHQOL can predict total mortality in males. Previous studies on HQOL, self-reported health and death, have found these variables to be correlated to each other only in males [38, 39]. Our results were consistent with the results of these studies. The relationship between biological sex and lifespan is explained by genes and sex hormones [40]. Males have a single X chromosome, resulting in a shorter lifespan than females with two X chromosomes, and the predominance of cancer in males is a result of sex-specific risk behaviors, sex hormones, and genetic pyro-programming of male cells [41]. With respect to lifestyle, males were found to consume more alcohol and smoke more often than females. These factors increase the risk of death from chronic heart disease and lung cancer [42]. Therefore, males are at higher risk of mortality and are more likely to be affected by OHQOL.

Gender differences have also been related to social background, healthcare-seeking behavior, and social support [43]. With respect to healthcare utilization, a notable tendency was observed among males to underestimate pain and illness and they were less inclined to use healthcare services and have regular medical checkups compared with females [41]. Nevertheless, females were found to self-report poor health more accurately than males on the HQOL index [39]. Similarly, females were more prophylactic than males, and therefore, were more sensitive to discomfort [44, 45]. Differences in life expectancies between the sexes may also be considered. It was suggested that the low mortality rate (mortality rate: among males 4.6%, females 3.7%) of female participants compared with that of male participants may have affected the predictive value. It has also been suggested that the average life expectancy of females compared with that of males may have influenced the results of this study. Given the age and health of the participants, there is also a concern that the average follow-up of 7.7 years may have not been long enough to examine deaths in females. The small sample size may also have affected the results, as the number of females was relatively small. Accordingly, in females, active use of health services may mitigate the impact of OHQOL.

After adjustment for the number of missing teeth in the multivariate analysis, low OHQOL was found to increase the risk of total mortality in our study. The number of missing teeth has been reported to be a cause of increased mortality because of the disruption of the occlusal status and decreased masticatory power, which is associated with decreased nutritional intake [46]. The number of missing teeth was a significant predictor of mortality [47]. Our findings suggest that OHQOL is independently associated with total mortality, regardless of the number of teeth. OHQOL includes social and psychological domains, in addition to physical function. OHQOL can be used to assess the comprehensive status of one’s mortality, as it evaluates the total impact on mortality, whereas other objective indices cannot. Further research and discussion based on this evidence in various settings are needed.

This study has several strengths, including the use of data obtained from subjective assessments by oral health professionals. The responses of the dentists could be considered more accurate than those of the general population. In addition, OHQOL was evaluated using one of the most widely used scales (GOHAI). To the best of our knowledge, this is the first study to analyze the association between OHQOL in dental professionals and total mortality.

However, certain limitations should be considered. First, information pertaining to the date and cause of death was obtained through the fraternal insurance program and the information pertaining to disabilities was insufficient. Therefore, the relationship with healthy life expectancy could not be investigated. Second, since the analysis was limited to total mortality, the cause of mortality was not specified. The oral health and OHQOL of the dentist population were better than those of the general population, but inter-individual variability was greater, as indicated by the standard deviation. However, since the questionnaire response rate was relatively low (32.9%) and OHQOL information was collected from only 20 prefectures, the generalizability of the results for Japanese dentists may be limited. The characteristics of participants and non-participants in a population-based cohort study were compared [48]. The participants were likely to have a higher social status, healthier lifestyles, and fewer instances of diseases compared to non-participants. The participants of this study may be healthier dentists with a lower risk of mortality than the general dentist population. If the study participants were indeed in better health, the impact of OHQOL may be attenuated by other factors that contribute to a reduced mortality risk. Third, since OHQOL was evaluated only once at baseline, changes in OHQOL over time could not be analyzed. We were not able to perform the analysis to consider all domains of HQOL, and further investigation of the relationship between OHQOL and HQOL is needed. Finally, findings from a very specific population of dentists would not be representative of the general population. We believe that the responses of dentists to GOHAI were sufficiently accurate. However, they may have been more cautious about their answers than the general population. Nonetheless, since the basic biological mechanism is the same for the human population, the association between OHQOL and the total mortality rate evident in dentists should also be applicable to the general population.

## Conclusions

An association between OHQOL and total mortality was observed among males of the dentist population in this study. A lower OHQOL could indicate an increased risk of total mortality. Evaluation of the oral health status using the OHQOL index is useful for screening potential factors that cannot be indicated by objective indices. Furthermore, OHQOL may be used as an indicator for selecting treatment plans and personalized care interventions, thus contributing to extending healthy life expectancy.

### Supplementary Information

**Additional file 1.** Baseline characteristics of male participants by GOHAI score quartile.

**Additional file 2.** Baseline characteristics of female participants by GOHAI score quartile.

**Additional file 3.** Total mortality aHRs based on male sex, according to the GOHAI score quartile encompassing General Health of HQOL data.

**Additional file 4.** Total mortality aHRs based on female sex, according to the GOHAI score quartile encompassing General Health of the HQOL data.

## Data Availability

For the informed consent requirements obtained from the participants, these data cannot be made publicly available. However, data are available from the corresponding author upon reasonable request.

## Abbreviations

aHRs: Adjusted hazard ratios
CI: Confidence interval
GH: General Health
GHQ-12: 12-Item General Health Questionnaire
GOHAI: General Oral Health Assessment Index
HQOL: Health-related quality of life
JDA: Japan Dental Association
LEMONADE: Longitudinal Evaluation of Multi-phasic, Odontological, and Nutritional Associations in Dentists
OHQOL: Oral HQOL
QOL: Quality of life
